# Warm Ether as an Anæsthetic

**Published:** 1887-11

**Authors:** 


### Warm Ether as an Anæsthetic.

Dr. M. W. Hobbes advocates, in the Cincinnati *Lancet-Clinic*, the advantage of warming ether previous to its administration in the production of anæsthesia. He and Dr. Taylor have tried the method in upward of thirty cases, and he writes that the patients not only come under the influence of the drug more readily, but they also recover more rapidly and pleasantly from the anæsthesia than patients generally do who have been brought under its influence in the ordinary way of administering ether cold.

